# Celluar Folding Determinants and Conformational Plasticity of Native C-Reactive Protein

**DOI:** 10.3389/fimmu.2020.00583

**Published:** 2020-03-31

**Authors:** Jian-Min Lv, Jun-Yao Chen, Zu-Pei Liu, Zhen-Yu Yao, Yue-Xin Wu, Cheng-Sen Tong, Bin Cheng

**Affiliations:** ^1^MOE Key Laboratory of Environment and Genes Related to Diseases, School of Basic Medical Sciences, Xi’an Jiaotong University, Xi’an, China; ^2^MOE Key Laboratory of Cell Activities and Stress Adaptations, School of Life Sciences, Lanzhou University, Lanzhou, China; ^3^Xi’an Key Laboratory of Children’s Health and Diseases, The Affiliated Children Hospital, Children’s Research Institute, Xi’an Jiaotong University, Xi’an, China

**Keywords:** protein folding, disulfide bond, C-reactive protein, inflammation, conformational activation

## Abstract

C-reactive protein (CRP) is an acute phase reactant secreted by hepatocytes as a pentamer. The structure formation of pentameric CRP has been demonstrated to proceed in a stepwise manner in live cells. Here, we further dissect the sequence determinants that underlie the key steps in cellular folding and assembly of CRP. The initial folding of CRP subunits depends on a leading sequence with a conserved dipeptide that licenses the formation of the hydrophobic core. This drives the bonding of the intra-subunit disulfide requiring a favorable niche largely conferred by a single residue within the C-terminal helix. A conserved salt bridge then mediates the assembly of folded subunits into pentamer. The pentameric assembly harbors a pronounced plasticity in inter-subunit interactions, which may form the basis for a reversible activation of CRP in inflammation. These results provide insights into how sequence constraints are evolved to dictate structure and function of CRP.

## Introduction

C-reactive protein (CRP) is a major human acute phase reactant mainly produced by hepatocytes as a pentamer. It is often used as a non-specific inflammation marker clinically, for its plasma level can immediately increase for more than 1,000 times upon tissue damage and infection (1–4). Moreover, as the first identified soluble pattern recognition receptor (PRR), CRP itself is directly involved in host defense, and inflammation (1–4). However, efficient folding mechanism of CRP in live cells remains unclear. Structurally, native CRP is composed of five identical, non-covalently assembled subunits (1–5). These subunits exhibit a flattened jellyroll topology consisting of a two-layered β sheet (strands A to M) (6). In our previous work, the structure formation of pentameric CRP has been demonstrated to proceed in a stepwise manner in live cells (7). Specifically, the N-terminal half of the two-layered β sheet, i.e., strands C to I termed the hydrophobic core (a.a. 32–111), and the a.a. 168–176 helix first fold spontaneously, leading to the formation of the intra-subunit disulfide bond (7). This event drives the following non-spontaneous stage of subunit folding, which involves a global conformation remodeling that requires the aid of cellular factors (7). Folded subunits then assemble into the native cyclic pentamer (7). However, it is unclear what sequence features underlie the stepwise pathway of folding and assembly of CRP in live cells.

Here, we identify critical sequence determinants for three key events, including the folding of the hydrophobic core, the formation of the intra-subunit disulfide bond, and the assembly of the pentamer. Our results further reveal the presence of a pronounced plasticity in pentameric assembly of CRP that underlies its conditional activation.

## Results and Discussion

### The Relatively Conserved P29/L30 Dipeptide in N-Terminal Is Critical for the Formation of the Hydrophobic Core

**FIGURE 1:**
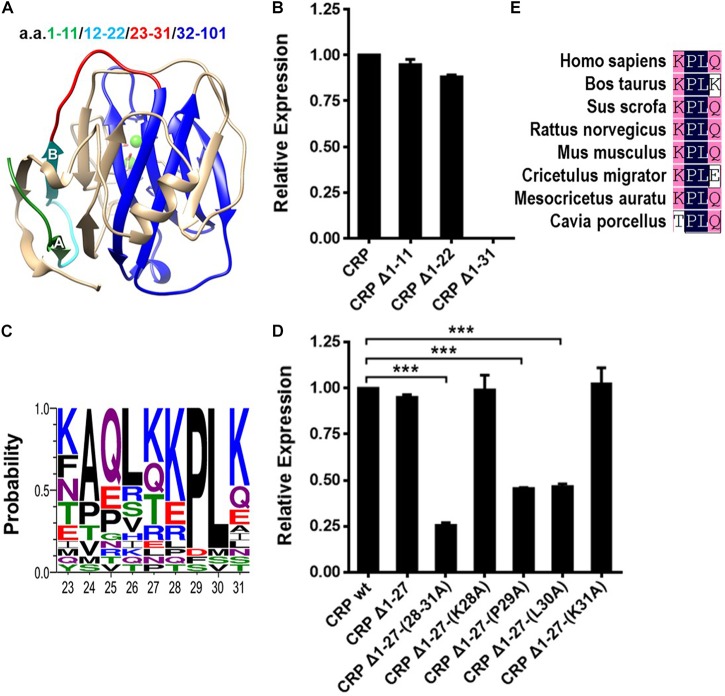
The conserved P29/L30 dipeptide in N-terminal is critical for folding of the hydrophobic core. **(A)** The subunit of CRP extracted from the pentameric crystal structure (PDB ID: 1B09). Segments within a.a. 1–31 are colored with different color for clarity. The N-terminal hydrophobic core consisting of a.a. 32–101 is colored blue. **(B)** CRP wildtype (wt) or mutants lacking the indicated sequences were expressed in the cytoplasm of *E. coli* cells. Their cytoplasmic levels were determined by immunoblotting with 3H12 mAb (7). Deletion of a.a. 1–31, but not a.a. 1–11 or a.a. 1–22, completely destabilized cytoplasmic CRP. **(C)** Conservation analysis of a.a. 23–31 of CRP from 21 species. KPLK (a.a. 28–31) emerges as the relatively conserved motif. **(D)** CRP wt and mutants lacking a.a. 1–27 or with point mutations of a.a. 28–31 were expressed in the cytoplasm of *E. coli* cells. Their cytoplasmic levels were determined by immunoblotting with 3H12 mAb (7). Simple truncation of a.a. 1–27 had little impact, while further mutations of the remaining residues 28–31 weakened the cytoplasmic stability in various degrees. Specifically, mutant Δ1-27-(28-31A), in which a.a. 28–31 were all replaced with alanine, exhibited a seriously impaired cytoplasmic stability. Moreover, the single point mutations of residues P29 and L30 also destabilized cytoplasmic CRP dramatically. **(E)** Conservation analysis revealed the PL dipeptide to be conserved in SAP from 8 species. ***p < 0.001.

a.a. 1–31 has been shown to be required for the spontaneous folding of the N-terminal hydrophobic core, as evidence by the fact that CRP mutant lacking this sequence showed marginal cytoplasmic stability and greatly impaired capacity to form intra-subunit disulfide bond (7). In the crystal structure, a.a. 1–31 is composed of two β-strands (A and B; a.a. 7–11 and 19–22) and three random coils without defined structures (a.a. 1–6, 12–18, and 23–31) (Figure 1A). Sequential truncations here revealed that the absence of a.a. 1–11 or 1–22 did not impair the cytoplasmic stability of non-secretory CRP in *E. coli* cells. By contrast, the mutant with truncation of a.a. 1–31 was nearly undetectable in the cytoplasm (Figure 1B). This would suggest that a.a. 23–31, the disordered coil most proximal to the hydrophobic core, instead of strands A and B, was sufficient to initiate the folding of strands C to I in the productive folding of the hydrophobic core.

In further analysis, we found that the KPLK motif in coil 23–31, i.e., a.a. 28–31, is relatively conserved among species (Figure 1C). Additional mutants were thus designed to test its potential effect (Figure 1D). Indeed, truncation of a.a. 1–27 did not impair the cytoplasmic stability of CRP subunit, supporting the importance of a.a. 28–31. Furthermore, alanine scanning of a.a. 28–31 precisely revealed P29 and L30 to be the most critical residues. Moreover, the PL dipeptide is also rather conserved in a paralog of CRP, i.e., serum amyloid P component (SAP) (6) (Figure 1E), highlighting its importance. Those evidences thus indicate that the conserved PL dipeptide might be the optimized motif evolved for rapid folding of the hydrophobic core and consequently the efficient production of CRP.

### A Single Residue in a.a. 168–176 Helix Determines the Intra-Subunit Disulfide Bonding

**FIGURE 2:**
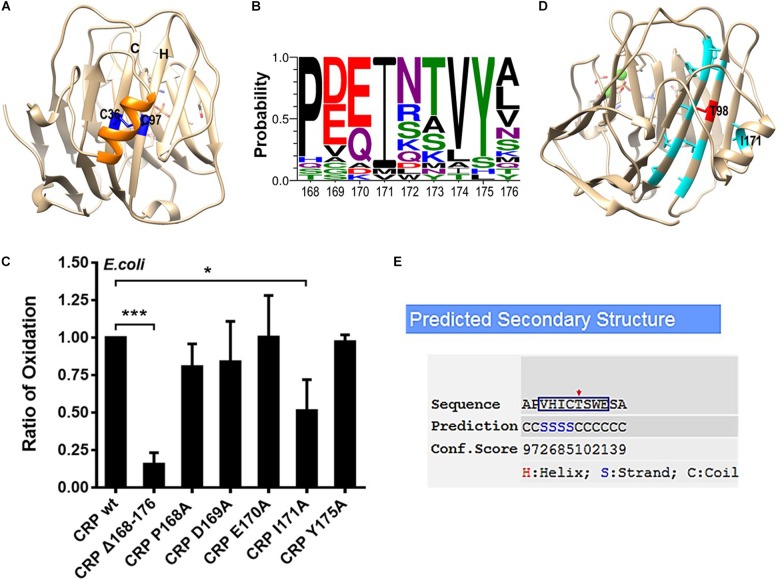
The efficient bonding of the intra-subunit disulfide depends on I171 within the covering helix. **(A)** The disulfide bond forming residues C36 and C97 in the subunit of CRP (PDB ID: 1B09) are colored as blue, while the covering helix a.a. 168–176 is colored as orange. **(B)** Conservation analysis of a.a. 168–176 of CRP from 21 species. P168, I171, and Y175 emerge as the most conserved residues. **(C)** CRP wildtype (wt) or mutants with a signal peptide of ALP were expressed in *E. coli*. The correct bonding of the intra-subunit disulfide bond in secreted CRP was determined by immunoblotting with 3H12 mAb (7). In addition to mutant lacking the entire covering helix, only I171A mutant significantly impaired the bonding of the intra-subunit disulfide. **(D)** Hydrophobic amino acids on strands C and H are colored as light blue while the hydrophilic T98 as red. The hydrophobic I171 points its side-chain to T98 directly. **(E)** The β-strand forming propensity of a.a. 94–101 (square), which constitutes strand H in the crystal structure of CRP, was calculated by I-TASSER (21, 22). T98 (red arrow) appears to be a strand-breaking residue being covered by I171. *p < 0.05; ***p < 0.001.

Following the formation of the hydrophobic core, a.a. 168–176 helix is folded and correctly positioned onto the core to induce the disulfide bonding between C36 on strand C and C97 on strand H (7) (Figure 2A). Sequence alignment revealed several highly conserved residues in a.a. 168–176 helix, of which P168, I171 and Y175 are most prominent (Figure 2B). Point mutations were thus made at these residues to examine their effects on intra-subunit disulfide bonding of secretory CRP. However, impaired bonding was only noted for I171A mutation (Figure 2C). I171 in the helix is positioned just over C97 and T98 of strand H. The hydrophilic T98 is actually incompatible with the sequence pattern of strand H, and could therefore be a strand-terminating residue (Figures 2D,E). In this regard, the covering of I171 would impose a niche of hydrophobicity and steric clash to enforce T98 to adopt a strand-compatible configuration, i.e., pointing its side-chain inward to the core. This may then stabilize strand H and its alignment with strand C to promote the bonding between C97 and C36. Additionally, it should be noted that although concurrent mutation of D169 and E170 has been shown in our previous work to prevent the formation of the intra-subunit disulfide bond (7), we found that mutating D169 or E170 alone had little effect. These suggest that concurrent mutation of D169 and E170 might disrupt the conformation of the helix and consequently affect the intra-subunit disulfide bonding.

### R118-D155 Salt Bridge Is Indispensable for Pentameric Assembly of CRP

**FIGURE 3:**
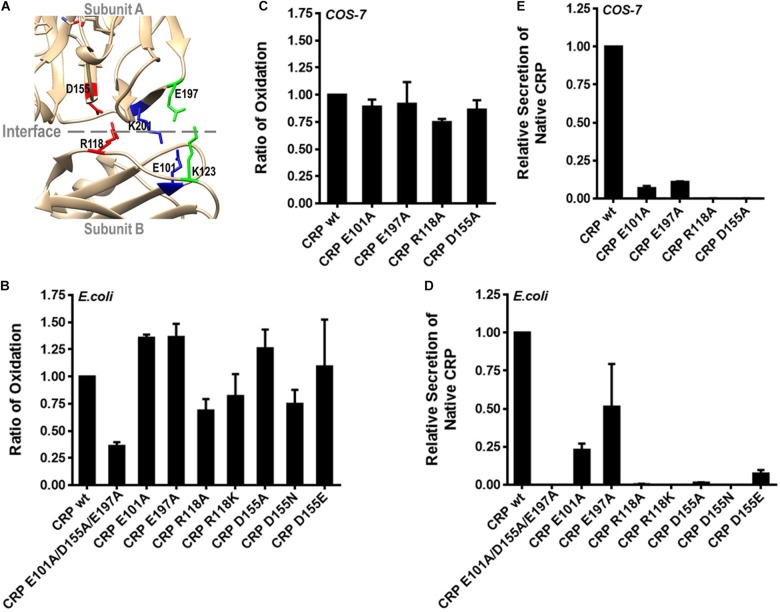
R118-D155 salt bridging mediates pentameric assembly of CRP. **(A)** The three inter-subunit salt bridges R118-D155, E101-K201 and K123-E197 of CRP are colored as red, blue and green, respectively. **(B)** CRP wildtype (wt) or mutants with a signal peptide of ALP or its own were expressed in *E. coli* or *COS-7* cells. **(B,C)** The correct bonding of the intra-subunit disulfide bond in secreted CRP was determined by immunoblotting with 3H12 mAb (7). The intra-subunit disulfide bond formed normally in CRP mutants with one of the salt bridges disrupted. **(D,E)** The secretion of pentameric CRP was determined by sandwich ELISA with 1D6 mAb (7). Only the disruption of R118-D155 salt bridge completely abrogated the secretion of pentameric CRP.

Pentameric CRP is assembled non-covalently by nearly folded subunits (7). The crystal structure of CRP suggests a critical role of three inter-subunit salt bridges, i.e., R118-D155, E101-K201 and K123-E197, in mediating pentamer assembly (Figure 3A) (6). We thus individually disrupted each of these salt bridges by mutations to clarify their relative importance. Disrupting single salt bridge did not profoundly affect subunit folding as the intra-subunit disulfide bonding of the corresponding mutants was largely undisturbed (Figures 3B,C). Rather, these mutants all exhibited defects in secretion of pentameric CRP by *E. coli* cells (Figure 3D). However, a complete loss of secretion was only observed for mutants with disrupted R118-D155 salt bridge. Similar results were also obtained with eukaryotic *COS-7* cells (Figure 3E). Therefore, although all three salt bridges contribute to the assembly of pentameric CRP, only R118-D155 salt bridge is indispensable. Accordingly, R118-D155 salt bridge is also conserved in mediating the pentameric assembly of SAP (6). It is thus likely that the formation of this salt bridge might be an early event licensing the subsequent assembly of the pentamer.

### Pentameric Assembly of CRP Exhibits Pronounced Plasticity

**FIGURE 4:**
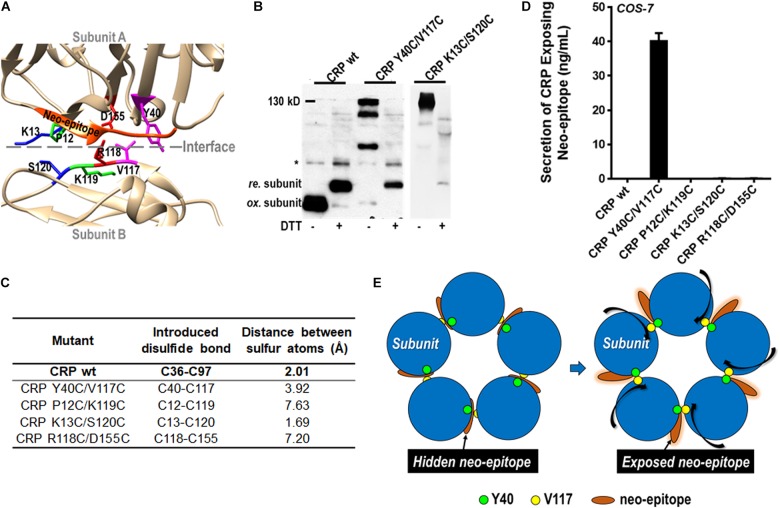
The pentameric assembly of CRP is adjustable. **(A)** The four residue pairs that were mutated to introduce inter-subunit disulfide bond are indicated with different color. The a.a. 199–206 neo-epitope is colored with orange. CRP wildtype (wt) or mutants with a signal peptide of its own were expressed in *COS-7* cells. **(B)** CRP secreted into conditioned media was examined by immunoblotting with 3H12 mAb (*non-specific signal). Mutants K13C/S120C and Y40C/V117C could be normally secreted as pentamer with inter-subunit disulfide bonds (∼130 kD). **(C)** The distances between the introduced cysteine residue pairs. **(D)** The neo-epitope exposure in secreted CRP was determined by ELISA with 3H12 mAb. The neo-epitope was exposed in Y40C/V117C, but not in K13C/S120C. **(E)** Proposed model of CRP activation by rotating subunits while maintaining the pentameric assembly.

Emerging evidence indicates that pentameric CRP can be activated by conformation changes upon interacting with factors enriched at inflammatory loci (3, 4, 8–11). One of the activated conformations has been suggested to maintain the pentameric assembly while showing a neo-epitope (a.a. 199–206) that is otherwise shielded by the native inter-subunit contacts (12–15). Such a conformation would imply that there is some degree of plasticity in the seemingly rigid assembly of pentameric CRP. The same implication has also been raised by comparing the pentameric structures of CRP and SAP (6). To directly test that implication, we introduced inter-subunit disulfide bonds between residues at different locations (Figure 4A) and expressed the corresponding mutants in eukaryotic *COS-7* cells with strict quality control on protein folding and secretion. The rationale is that if a mutant can be secreted as a pentamer with inter-subunit disulfide bonds formed between residues at a distance longer than that of a typical disulfide bond, it would be an indication of plasticity in pentameric assembly.

Of the tested mutants (including the one based on R118-D155 salt bridge), only K13C/S120C and Y40C/V117C were found to be secreted with inter-subunit disulfide bonds (Figure 4B). The distances between the sulfur atoms of induced cysteines in K13C-S120C and Y40C-V117C are ∼1.7 Å and ∼3.9 Å, respectively; while that of a typical disulfide bond is ∼2.0 Å (Figure 4C). Therefore, no significant remodeling in the native pentameric assembly would be required for the formation of the inter-disulfide bond in K13C/S120C. By contrast, a significant remodeling would be anticipated in case of Y40C/V117C. In line with that speculation, the neo-epitope of a.a. 199–206 was indeed exposed in Y40C/V117C but not in K13C/S120C (Figure 4D). The distances between other mutated residue pairs were over 7 Å, and their failure of secretion probably reflect an upper limit of the plasticity for the pentameric assembly of CRP. These results together suggest a mode of CRP activation by rotating subunits while maintaining the pentameric assembly (Figure 4E).

In summary, the present work has identified sequence determinants that are key to the success of CRP’s cellular folding and assembly. The identified determinants act in both spontaneous and non-spontaneous phases of CRP structuring in live cells, and their actions cannot be compensated by cellular factors assisting folding. These findings thus highlight that structure is intrinsically encoded by sequence, but it remains to be elucidated how the sequence determinants orchestrate to dictate the final structure of CRP. Interestingly, the pentameric assembly of CRP can be moderately adjusted, thereby allowing the exposure of functional epitope with the native subunit conformation largely unaltered. This may provide the structural basis for a reversible activation of CRP at inflammatory loci.

## Materials and Methods

### Materials

CRP purified from human ascites (purity > 97%) were purchased from the BindingSite (Birmingham, United Kingdom; catalog number: BP300.X). 1D6 and 3H12 monoclonal antibodies (mAbs) against CRP were prepared as described (16). 1D6 reacts exclusively with native CRP, whereas 3H12 recognizes a neo-epitope exposed only in dissociated subunits (16).

### Construction and Expression of Mutants

C-reactive protein wildtype or mutants were constructed and expressed in *E. coli* (BL21) or *COS-7* cells as described in our previous work (7). Briefly, for expression in *E. coli* (BL21) cells, the coding sequence of wild-type and mutant CRP were cloned into pET42c plasmids. Where necessary, the N-terminus of CRP was fused with signal peptide of alkaline phosphatase (ALP) to enable secretion. The transformed BL21 cells were cultured and then induced with 0.5 mM IPTG for 24 h at 16°C. The cultures were finally centrifugated at 5,000 *g* for 5 min to collect the supernatants and pelleted cells. For expression in *COS-7* cells, coding sequences of wild-type and mutant CRP with its own signal peptide were cloned into pcDNA3.1 plasmids (Invitrogen). *COS-7* cells were transfected with the constructed plasmids by Lipofectamine 2,000 (Invitrogen) and then cultured for 48 h before sample collection.

### Characterization of CRP Folding in Live Cells

The cellular stability and intra-subunit disulfide bonding of CRP were examined by immunoblotting, wherein reduced subunits ran slower than oxidized counterparts (7, 17–19). To keep the original redox state, N-ethylmaleimide (NEM; 5 mM) was added upon cell lysis or sampling of conditioned media to protect free cysteines from oxidation. The levels of secreted CRP in conditioned media were determined with conformation-specific, sandwich ELISA (7, 20). A sheep anti-human CRP polyclonal antibody (BindingSite; 5 μg/ml) was immobilized onto microtiter wells as the capture antibody, whereas 1D6 and 3H12 mAbs were used as the detection antibody.

### Statistical Analysis

Data were obtained from at least three independent experiments and represented as mean ± SEM. Statistical analysis was performed by two-tailed Student’s *t*-test, one-way ANOVA with Tukey *post hoc* or Kolmogorov-Smironv tests as appropriate. Values of *p* < 0.05 were considered significant.

## Data Availability Statement

All datasets generated for this study are included in the article/supplementary material.

## Author Contributions

J-ML designed the research. J-YC, Z-PL, Z-YY, Y-XW, C-ST, and BC performed the experiments. J-ML, J-YC, and Z-PL analyzed the data. J-ML wrote the manuscript. All authors reviewed the results and approved the final version of the manuscript.

## Conflict of Interest

The authors declare that the research was conducted in the absence of any commercial or financial relationships that could be construed as a potential conflict of interest.
